# Evaluation of Phytochemical, Antioxidant, and Memory-Enhancing Activity of *Garuga pinnata* Roxb. Bark and *Bryophyllum pinnatum* (Lam) Oken. Leaves

**DOI:** 10.1155/2021/6649574

**Published:** 2021-04-27

**Authors:** Ravin Bhandari, Sabina Gyawali, Nisha Aryal, Devika Gaire, Kalpana Paudyal, Abaru Panta, Pooja Panth, Dirgha Raj Joshi, Rabindra Kumar Rokaya, Pramod Aryal, Jitendra Pandey

**Affiliations:** ^1^Department of Pharmacy, Crimson College of Technology, Affiliated to Pokhara University, Devinagar-11, Butwal 32900, Nepal; ^2^Department of Pharmacology, Karnali Academy of Health Science, Jumla, Chandannath 21200, Nepal

## Abstract

*Garugapinnata* Roxb. (Burseraceae) is a medium-sized tree widely available all over the tropical regions of Asia. *Bryophyllum pinnatum* (Lam) Oken. (Crassulaceae) is an indigenous and exotic plant grown in tropical regions. Both plants have been used for their anti-inflammatory, antioxidant, anticancer, wound healing, antidiabetic activities, etc. This investigation was designed to explore the result shown by methanolic extract of *Garuga pinnata* bark and *Bryophyllum pinnatum* leaves, on cognitive power and retention of the memory in experimental mice along with quantification of phenolic compounds and DPPH radicals neutralizing capacity. The memory-enhancing activity was determined by the elevated plus-maze method in Scopolamine-induced amnesic mice, using Piracetam as allopathic and Shankhpushpi as ayurvedic standard drugs. Two doses (200 and 400 mg/kg p.o.) of both extracts were administered to mice up to 8 consecutive days; transfer latency of individual group was recorded after 45 minutes and memory of the experienced things was examined after 1 day. DPPH assay method and the Folin–Ciocalteu method were employed to determine antioxidant potency and total phenol amount, respectively. 400 mg/kg of the methanolic *B. pinnatum* bark extract significantly improved memory and learning of mice with transfer latency (TL) of 32.75 s, which is comparable to that of standard Piracetam (21.78 s) and Shankhpushpi (27.83 s). Greater phenolic content was quantified in *B. pinnatum* bark extract (156.80 ± 0.33 *µ*g GAE/mg dry extract) as well as the antioxidant potency (69.77% of free radical inhibition at the 100 *µ*g/mL concentration). Our study proclaimed the scientific evidence for the memory-boosting effect of both plants.

## 1. Introduction

Memory is a vital process by which experiences are recorded and can be used to adapt their responses to the environment. Alzheimer's disease (AD) is a major condition related to memory impairment and it can be characterized as a continuous neurodegenerative disorder that can lead to senile dementia, particularly in the cortex and hippocampus region. Besides distinct clinical manifestations, behavioral hallmarks involve progressive loss in memory, judgment capacity impairment, decision-making problem, physical atmosphere orientation, and language problem [1]. Studies of patients with AD revealed the decreasing levels of acetylcholine in the brain [2]. Although there are many treatment options available to treat AD, due to the lack of complete efficacy and side effects, the interest in plant-based herbal medicine is increasing nowadays.

Shankhpushpi is a widely used herbal drug in the management of cognitive disorders, from the times of Acharya Charak (200 BC) to date [3]. Shankhpushpi has been categorized as a Medhya Rasayana, that is, an efficient brain tonic by Ayurvedic texts. According to the Ayurvedic Pharmacopeia of India, the whole parts of *Convolvulus pluricaulis* Choisy are approved for medicinal uses [4]. Based on their potentiality to treat memory-related dysfunctions and shape of the flower, the other three botanicals, namely, *Clitoria ternatea* Linn., *Canscora decussata* Schult., and *Evolvulus alsinoides* Linn. are also categorized as Shankhpushpi by Indian practitioners [5]. It is most commonly indicated for anxiety, depression, mental illness, seizure, sleeplessness, burning pain, hyperlipidemia, urinary complications, and edema. It has been reported that Shankhpushpi regulates epinephrine and hydrocortisone synthesis from the body [4, 6]. Shankhpushpi increases the acetylcholine amount in the hippocampus region depending upon the dose administered that might contribute to its memory enhancement activity [7]. Neutralization of neurotoxic free radicals along with enhancement of the neurite outgrowth also plays a vital role in this effect [8, 9].

*Bryophyllum pinnatum* (Crassulaceae) is abundantly distributed in the tropical and subtropical regions around the world. Aerial parts or entire plant can be utilized to manage hypertension, syphilis, jaundice, candidiasis, dysmenorrhea, convulsions, and so forth. Many bioactive compounds, like bryotoxin A, B, C, caffeic acid, and protocatechuic acid, have been reported from this plant [10] that exhibit various activities like antioxidant, antiulcer, analgesic activity [11]; antiurolithiasis; and so forth [12].

*Garuga pinnata* (Burseraceae) is a deciduous plant found in India and east and south parts of Asia. It has been reported that *G. pinnata* extracts exhibited antiulcer, anticancer, antibacterial, antidiabetic, and antioxidant activity [13]. Ethnomedicinally, in Nepal, decoction from the leaf of *B. pinnatum* and bark of *G. pinnata* is used by the ethnic groups of the Tharu community to enhance memory. However, there is no scientific evidence on this ethnomedicinal claim. Thus, our study is mainly focused on investigating the memory-improving effect of these two plants along with their antioxidant activity.

## 2. Materials and Methods

### 2.1. Chemicals

The chemicals used are Piracetam and Scopolamine hydrobromide (Asian Pharmaceuticals, Nepal), Shankhpushpi (Bhagvati Herbal and Healthcare Private Limited, India), ascorbic acid (Merck), DPPH (Sigma-Aldrich), sodium bicarbonate (Fisher Scientific), hydrochloric acid, mercuric chloride, potassium chloride, ferric chloride, lead acetate, and Kagnesium ribbon (SD fine-chem limited).

### 2.2. Plants Collection and Extraction

*G. pinnata* and *B. pinnatum* were collected from Boadgoau, Kapilvastu, Nepal (27.5518°N, 83.0469°E, 107 m altitude from sea level) during September 2019 and were identified by National Herbarium and Plant Laboratories, Nepal. The herbarium voucher specimens of the plants have been deposited in the pharmacognosy laboratory of Crimson College of Technology (CCT-HRB-075-187 (*G. pinnata*) and CCT-HRB-075-188 (*B. pinnatum*)). Bark and leaves of both plants were shade dried; comminuted to fine powder and extraction was carried out by the triple maceration process using methanol as the menstruum. The extracts were dried with the help of the rotary vacuum evaporator at 45°C and sticky dried extract was obtained which was further stored at 4°C until use.

### 2.3. Phytochemical Screening

Preliminary screening for the presence of secondary metabolites in the sample was performed by using previously described methods with slight modifications [14–16].

### 2.4. Total Phenol Content (TPC)

For the determination of TPC, the Folin–Ciocalteu method was adopted [17]. TPC was calculated from the calibration curve of the Gallic acid and presented in the terms of *μ*g gallic acid equivalent (GAE)/mg extracts.

### 2.5. Antioxidant Potency Measurement

The potency of the plant extracts to neutralize the DPPH free radicals was determined by the previously established method [18]. Ethanol and ascorbic acid were used as the blank sample and standard sample, respectively. The percentage of radical scavenged was calculated from the following equation:

(1) $$\begin{array}{c} \text{percentage of radical neutralized}=\left[\left(A0\;-\;A1\right)/A0\right]*100\%\;. \end{array}$$

Here, *A*_0_ denoted the DPPH solution absorbance and *A*_1_ denoted the test sample absorbance.

### 2.6. Animal's Ethical Approval and Acute Toxicity Studies

Male and female Swiss albino mice (25–30 g) were placed inside polypropylene cages with standard feed and water ad libitum. Animal handling, care, and experimental design were conducted according to the guidelines of the Institutional Review Committee, Nepal Health Research Council (Ref. no. 622/2019). Experimental animals were acclimatized for two weeks before experimental procedures [19–21]. Guidelines given by the Organization of Economic Cooperation and Development were employed to study acute toxicity in the experimental animal [22].

### 2.7. Memory-Enhancing Activity

#### 2.7.1. Elevated Plus-Maze Method

Elevated plus-maze and Scopolamine-induced amnesia were used for the exteroceptive and interoceptive behavior design, respectively, to monitor the cognitive power and retention of the memory, according to a previously established method with slight modification [23].

The elevated plus-maze having 2 open arms (16 × 5 centimeters), 2 closed arms (16 × 5 × 12 centimeters) raised vertically with the 25-centimeter height was applied. Individual experimental animals were kept at the edge of the open arm facing far from the middle rostrum; then, total duration for moving from the edge of the open arm to both closed arms using all legs (Transfer latency, TL) was noted. Those animals that failed to reach inside any closed arm in a period of 90 sec were kindly propelled toward closed arms and TL was considered to be 90 s. The mice were permitted to search the maze for the next 10 s and afterward come back to the cage. Memory of this experienced work was investigated 1 day after the initial examination day.

Transfer latency after 1 day was interpreted in the terms of Inflection Ratio (IR), by applying the following equation:

(2) $$\begin{array}{c} \text{IR}=\left(L1\;–\;L0\right)/\;L0, \end{array}$$

where *L*_0_ is the transfer latency after 1 day and *L*_1_ is the first transfer latency expressed as seconds.

#### 2.7.2. Experimental Design

A total of 48 mice were classified into 8 groups (*n* = 6). All groups except for vehicle control (Group I) were treated with Scopolamine hydrobromide (0.4 mg/kg) intraperitoneally, on the eighth day of extract/standard drug treatment to induce amnesia. Transfer latency was recorded after 45 minutes and the memory of experienced things was evaluated after 1 day in every classification:

1. Group I : vehicle control
2. Group II : Scopolamine control
3. Group III : *B. pinnatum* leaves test sample (200 mg/kg)
4. Group IV : *B. pinnatum* leaves test sample (400 mg/kg)
5. Group V : *G. pinnata* bark test sample (200 mg/kg)
6. Group VI : *G. pinnata* bark test sample (400 mg/kg)
7. Group VII : Shankhpushpi (5-fold dilution)
8. Group VIII : Piracetam (100 mg/kg)

### 2.8. Statistical Analysis

All the data were presented in terms of average ± SD. The mean differences between the different classes were calculated with the help of one-way ANOVA (MS-Excel 2019) and considered significant if *p* < 0.05.

## 3. Results

### 3.1. Extractive Yield, Phytochemical Screening, and Total Phenolic Content

**Figure 1:**
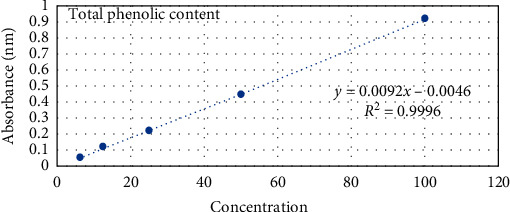
Calibration curve of gallic acid standard for total phenolic content. Concentrations of the gallic acids were prepared in *µ*g/mL.

Extractive yield for the methanolic extract of *G. pinnata* bark and *B. pinnatum* leaves was 12.04 and 10.46%, respectively. Qualitative phytochemical examination revealed the occurrence of phenolic compounds, flavonoids, tannins, saponin, and alkaloids. The entire phenolic amount was quantified by utilizing the calibration curve of the standard gallic acid (Figure 1) and interpreted as gallic acid equivalent (GAE). The total phenolic content of *B. pinnatum leaves* and *G. pinnata* bark was found to be 27.782 ± 0.25 and 156.80 ± 0.33 *µ*g GAE/mg dry extract, respectively.

### 3.2. Antioxidant Activity

According to Table 1, the DPPH radicals neutralizing activity shown by the methanolic extract of *G. pinnata* bark (MGPB) (EC_50_ 50.14 *μ*g/mL) and methanolic extract of *B. pinnatum* leaf (MPBL) extract (EC_50_ 109.41 *μ*g/mL) revealed that they have a moderate antioxidant capacity in comparison to ascorbic acid standard (EC_50_ 5.47 *μ*g/mL). Furthermore, the activity depends upon the dose used. The free radical neutralizing capacity of MGBP is greater as compared to the MBPL.

### 3.3. Acute Toxicity Test

After the continuous monitoring of MBPL and MGPB oral ingestion at different doses (0.2, 0.4, 0.8, and 1 g/kg), it was concluded that plant extracts have no acute toxicity.

### 3.4. Memory-Boosting Effect

**Figure 2:**
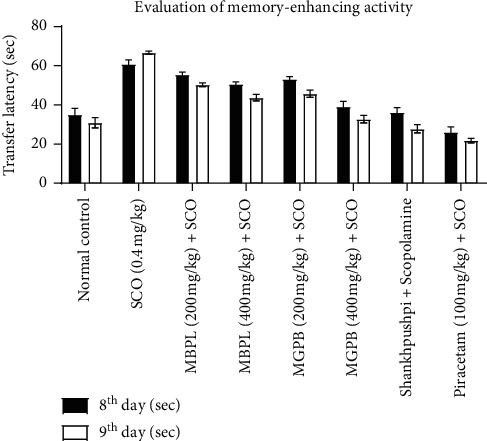
Bar diagram comparing the memory-enhancing activity of *B. pinnatum* leaves and *G. pinnata* barks with standard drug Piracetam and Shankhpushpi in the terms of transfer latency (SCO: Scopolamine, MBPL: *Bryophyllum pinnatum* leaves methanol extract, MGPB: *Garuga pinnata* bark methanolic extract).

We verified the memory-enhancing activity of the two ethnomedicinally claimed plants for the first time. Oral ingestion of the MBPL (200 mg/kg) for 8 consecutive days did not produce any prominent result on TL, on the 8^th^ and 9^th^ day whereas, at the higher dose (400 mg/kg), it exhibited an incredible reduction in TL (*p* < 0.05, *p* < 0.005), on the 8^th^ and 9^th^ day, suggesting noteworthy enhancement in cognitive and remembrance power (Table 2 and Figure 2). MGDP extract at both doses exhibited remarkably higher memory-enhancing activity (*p* < 0.005) on the 8^th^ and 9^th^ day. Scopolamine hydrobromide (0.4 mg/kg, i.p.) injected on the 8^th^ and 9^th^ days has high TL, indicating retardation in cognitive and remembrance power. Oral administration of the MBPL and MGPB at optimum doses (200 and 400 mg/kg up to 8 regular days) effectively improved Scopolamine produced memory loss. The results obtained from the plant extracts are comparable to the standard Piracetam and Shankhpushpi. However, Shankhpushpi showed better results than all the plant extracts.

## 4. Discussion

Preliminary screening of phytochemicals is an important step for detecting various bioactive secondary metabolites of plants, having a vital role towards beneficial medicinal and physiological activities such as antioxidant, antidiabetic, and anticancer activities [24]. Our study proclaimed the occurrence of bioactive compounds such as flavonoids, alkaloids, phenols, tannins, and saponins, which are similar to the results of another study for both plants [25, 26]. Phenolic compounds display potent antioxidant effects by scavenging free radicals due to the presence of hydroxyl groups. Hence, quantitative phenolic content determination gives a clear idea about the antioxidant potency of plants and their biological activities. Greater phenolic content in the bark of *G. pinnata* (156.80 ± 0.33 *µ*g GAE/mg) than in *B. pinnatum* leaves (27.782 ± 0.25 *µ*g GAE/mg) reported in our study correlates the greater antioxidant activity observed and corresponds to the previous findings [27, 28]. Plant-derived free radical scavengers such as flavonoids and phenolic compounds are responsible for antioxidant activity [29].

DPPH free radical inhibition percentage data from Table 2 demonstrates that methanolic extract of *G. pinnata* bark is more effective in inhibiting DPPH free radical compared to methanolic extract of *B. pinnatum* in all the tested concentrations. The higher amount of phenolic compounds might be the reason for better antioxidant potency of the *G. pinnata* bark extract. In a previous study, 40% of DPPH free radicle was found to be inhibited by 100 µg/mL of methanolic *B. pinnatum* extract [30]. In another study, the minimum effective concentration for ethanolic extract was found to be 144.09 µg/mL [31].

An elevated plus-maze model was adopted to determine the memory-enhancing efficacy of plant extracts in which Scopolamine was injected to induce amnesia in mice [23]. However, continued administration of both extracts facilitated better improvement of the memory acquisition and retention (proportional to the amount of dose administered) as compared to animals treated with Scopolamine, thus verifying nootropic effect [32]. In an elevated plus-maze model, reduced TL value suggested the upgrade of the cognitive power and vice versa. Recent studies have identified that this is an accepted model to study the learning and memory process in mice [33]. Immunological studies have shown that neuronal cell damage caused by inflammation can induce AD and prolonged use of anti-inflammatory agents can reduce the severity of this disease by inhibiting onset and the progression of AD [1, 23]. Thus the anti-inflammatory activity of *G. pinnata and B. pinnatum* might be a major contributing factor for observed memory-enhancing activity [34, 35]. Oxygen-free radicals can generate neurotoxin and may act as a causative factor for the AD. The memory-boosting potency of both plants may be accredited to their free radical neutralizing capacity due to which liable neurons get affected by a lower amount of toxic free radical resulting in decreased neuronal toxicity and enhanced brain activity, thus boosting the cognitive power [23]. Also, the phenolic compounds can trigger the specific intracellular signaling pathway to improve memory process [36]. Consequently, higher phenolic content and antioxidant activity of the *G. pinnata* may have contributed to its higher memory-enhancing effect as compared to *B. pinnatum* in this study. In a previous study on the Swiss albino mice model, an aqueous extract of *B. pinnatum* (AEBP) at 200 mg/kg body weight had significantly controlled the sleeping time and other overall behaviors, induced by phenobarbitone along with delaying effect on the onset of picrotoxin and strychnine induced convulsion. *B. pinnatum* extract might exert depressive effects on the central nervous system as a result of its GABAergic and less commonly glycinergic transmission [37]. Memory-enhancing effect of *B. pinnatum* was also supported by a previous study, in which AEBP leaves had shown improvement of short-term memory loss by suppressing CCL_4_ induced acetylcholinesterase activity. The leaf extract was very much prominent to reduce CCL_4_ induced nitric oxide (NO) and malondialdehyde (MDA) overproduction in the brain to protect from oxidative stress. Increased MDA level is an indication of oxidative stress to result in oxidative damage of the brain. Oversynthesis of NO diminishes the function of antioxidant enzymes, hence leading to neurodegenerative diseases such as AD and depression [38]. Acetylcholine (ACh) is the major neurotransmitter of the cholinergic system associated with cognitive functions such as episodic and spatial memory, learning behavior, working memory, learning, and cerebral blood flow regulation [39]. Acetylcholine (ACh) concentration is controlled by enzyme acetylcholinesterase (AChE) which is capable of hydrolyzing this neurotransmitter. An increase in the concentration of AChE in the brain results in the degradation of Ach, eventually reducing the ACh receptors and then inducing undesirable effect on cholinergic neurotransmission, and also results in impairment of cognitive function as well as other neurological disorders [40]. Administration of AEBP leaves in the dose of 25 and 50 mg/kg body weight had resulted in prominent inhibition in AChE activity, in comparison to the negative control group and also it had resulted in a significant decrease in adenosine deaminase (ADA) concentration in the brain of albino mice. It was proven that increasing concentration of ACh in the synaptic cleft facilitates the cognitive functions, such as learning and memory, and modulation of cerebral blood flow. Increasing concentration of ADA is associated with the declination of neuron protective endogenous molecule adenosine [38, 39]. The barks of *G. pinnata* contain different types of diarylheptanoids [41, 42], which are effective in neuron protection and cognition enhancement. These compounds can remarkably improve the amnesia, induced by scopolamine, in passive avoidance tests. The diarylheptanoids can significantly increase phosphorylation of the cyclic AMP response element-binding protein (CREB) and expression of brain-derived neurotrophic factor (BDNF) in the hippocampus and cortex of the scopolamine-treated mice. They also notably protected hippocampal HT22 cells from glutamate insult-induced neurotoxicity. Overall, diarylheptanoids may mitigate memory deficiency by activating the BDNF–CREB pathway and inhibit neuronal cell death to prevent neurodegeneration [43, 44]. Besides, leaves of *B. pinnatum* and bark of *G. pinnata* were reported to present potent anti-inflammatory nonsteroidal derivatives [10], phytosterols, triterpenoids, alkaloids, glycosides, polyphenol, *β*-amyrin structure, and so on [34, 35, 45, 46]. Flavonoids are present abundantly in both plants and several studies show that natural flavonoids can produce an anxiolytic effect without inducing sedation and amnesia [47, 48]. Similarly, terpenoids are known to cause sedative and anxiolytic effects [32]. The precise mechanism of action remains to be explained but our findings are important as they validate one of the folk uses of the *G. pinnata* bark and *B. pinnatum* leaves as a medicinal plant in Nepal.

## 5. Conclusion

*G. pinnata* and *B. pinnatum* both contain bioactive phytoconstituents like alkaloids tannins, phenols, and flavonoids with free radical scavenging and memory-boosting ability. So, it is possible to utilize these plants for the management of cognitive impairment, related to AD. Besides, the significant memory-enhancing activity of these plants may be associated with their phenolic content and antioxidant potency. Further study is needed at the preclinical and clinical level with the elucidation of the exact mechanism.

## Figures and Tables

**Table 1:** Antioxidant activity of leaves of *G. pinnata* and barks of *B. pinnatum*. MGPB has significant antioxidant activity compared to MBPL in a dose-dependent manner.

Conc. (*µ*g/mL)	MBPL	Percentage inhibition	
		MGPB	AA
0.1	17.69 ± 0.58	24.07 ± 0.123^*∗*^	34.76 ± 4.5^*∗∗*^
1	22.35 ± 0.11	28.74 ± 0.92^*∗*^	42.48 ± 3.51^*∗∗*^
10	23.83 ± 0.30	40.78 ± 0.67^*∗*^	88.36 ± 0.85^*∗∗*^
100	47.42 ± 0.69	69.77 ± 0.43^*∗*^	95.25 ± 1.44^*∗∗*^

MBPL : methanolic extract of *B. pinnatum* leaf, MGPB : methanolic extract of *G. pinnata* bark, and AA : ascorbic acid. ^*∗*^*p* < 0.05 and ^*∗∗*^*p* < 0.01 when correlated to MBPL (note: all the tests were done in triplicate).

**Table 2:** Memory-enhancing activity of plant extract.

Group	(TL) 8^th^ day (s)	(TL) 9^th^ day (s)	IR
Vehicle control (saline)	35.05 ± 3.17	30.96 ± 2.64	0.132 ± 0.288
Scopolamine (0.4 mg/kg)	60.74 ± 2.27	66.67 ± 0.76	0.88 ± 0.028
MBPL (200 mg/kg) + Scopolamine	55.36 ± 1.37^*∗∗*^	50.3 ± 0.99^*∗∗*^	0.1 ± 0.073
MBPL (400 mg/kg) + Scopolamine	50.5 ± 1.34^*∗∗*^	43.67 ± 1.69^*∗∗*^	0.156 ± 0.10
MGPB (200 mg/kg) + Scopolamine	53.12 ± 1.3^*∗*^	45.67 ± 1.85^*∗∗*^	0.163 ± 0.061
MGPB (400 mg/kg) + Scopolamine	39.16 ± 2.69^*∗∗*^	32.75 ± 1.89^*∗∗*^	0.194 ± 0.2147
Shankhpushpi + Scopolamine	36.16 ± 2.51^*∗∗*^	27.83 ± 2.1^*∗∗*^	0.299 ± 0.141
Piracetam (100 mg/kg) + Scopolamine	26.12 ± 2.6^*∗∗*^	21.78 ± 1.19^*∗∗*^	0.19 ± 0.611

MBPL and MGBP at both tested doses significantly reversed memory impairments induced by Scopolamine and are comparable to that of both controls. MGPD (400 mg/kg) exhibited the best memory-enhancing activity among all test samples, with minimum transfer latency time. All the data are calculated as average ± SD; *n* = 6. Data are statistically effective at ^*∗*^*p* < 0.05 and highly effective at ^*∗∗*^*p* < 0.005 in comparison to Scopolamine.

## Data Availability

All the data used to support the result of this research are available from R. Bhandari upon request.
